# Paclitaxel Drug-Coated Balloon (Optilume^®^) for Bladder Neck Stenosis and Vesicourethral Anastomotic Stenosis: A Narrative Review

**DOI:** 10.3390/medicina62050898

**Published:** 2026-05-06

**Authors:** Tomasz Ufniarski, Mikołaj Frankiewicz, Maja Frankiewicz, Marcin Matuszewski

**Affiliations:** 1 Urology Clinic, University Clinical Centre, 80-952 Gdańsk, Poland; 2 Department of Urology, Medical University of Gdańsk, 80-210 Gdańsk, Poland; 3 Department of Pharmaceutical Technology, Medical University of Gdańsk, 80-210 Gdańsk, Poland

**Keywords:** drug-coated balloon, Optilume, bladder neck stenosis, vesicourethral anastomotic stenosis, paclitaxel, urethral stricture, posterior urethral stenosis, recurrence

## Abstract

*Background and Objectives*: Bladder neck stenosis (BNS) and vesicourethral anastomotic stenosis (VUAS) are challenging complications following prostate surgery and radiation therapy, with recurrence rates reaching 30–60% after conventional endoscopic management. The Optilume^®^ paclitaxel drug-coated balloon (DCB) has emerged as a novel minimally invasive treatment combining mechanical dilation with local anti-fibrotic drug delivery. This narrative review synthesizes current evidence on Optilume DCB specifically for BNS and VUAS. *Materials and Methods*: A comprehensive literature search identified eight relevant publications (2024–2026), including randomized controlled trials, prospective and retrospective cohort studies, and case series addressing Optilume DCB for posterior urethral stenoses. *Results*: Across the reviewed studies, freedom from reintervention for BNS ranged from 77.5% to 100% at 12 months, while VUAS outcomes were more variable (40–81%). A comparative study of 141 patients demonstrated significantly improved recurrence-free survival with DCB versus standard endoscopic treatment (HR 0.40, *p* = 0.021). Radiation-induced posterior urethral stenosis showed 81.1% freedom from repeat intervention. Complications were predominantly minor (Clavien-Dindo grade I), with no de novo incontinence attributable to the device. *Conclusions*: Optilume DCB represents a promising minimally invasive option for BNS and VUAS, particularly in patients with recurrent disease or those unsuitable for reconstructive surgery. BNS appears to respond more favorably than VUAS, likely reflecting distinct pathophysiological mechanisms. Prior radiation therapy remains a negative prognostic factor. Prospective randomized trials with longer follow-up are needed to define the role of DCB in posterior urethral stenosis management.

## 1. Introduction

Bladder neck stenosis (BNS) and vesicourethral anastomotic stenosis (VUAS) represent challenging iatrogenic complications of prostate surgery and radiation therapy. BNS occurs in 0–10% of patients following transurethral procedures for benign prostatic hyperplasia (BPH), with rates as high as 31% after high-intensity focused ultrasound (HIFU) [1]. VUAS develops in 6–15% of patients after open radical prostatectomy and 0–3% following robot-assisted radical prostatectomy (RARP), with salvage prostatectomy carrying a risk of up to 40% [2]. These conditions share a common endpoint of fibrotic luminal narrowing but differ substantially in pathophysiology, tissue characteristics, and treatment responsiveness from anterior urethral strictures [3,4].

The pathophysiology of BNS involves excessive scar formation and fibrosis at the bladder neck, driven by surgical trauma, aggressive fulguration with high ablative energy, and extensive bladder neck resection [5,6]. VUAS, by contrast, results primarily from ischemia at the vesicourethral anastomosis, compounded by tension on the surgical anastomosis and, in many cases, antecedent or adjuvant radiation therapy [3,7]. Unlike anterior urethral strictures—which typically arise from spongiofibrosis within the corpus spongiosum due to trauma, instrumentation, or lichen sclerosus—BNS and VUAS involve fibrotic contracture of a surgical defect in the absence of a normal urethral wall, making them inherently more resistant to endoscopic management [3,4,8].

Conventional management of BNS and VUAS relies on endoscopic dilation and direct vision internal urethrotomy (DVIU), but recurrence rates remain high, reaching 27–30% at one year and exceeding 55–60% for VUAS after transurethral resection [4,9]. Adjunctive therapies including mitomycin C injection, corticosteroid injection, and temporary stent placement have been explored, but none have demonstrated consistently durable outcomes [3,4]. Urethroplasty, while offering success rates of 70–95%, is associated with significant morbidity, including a 19–35% risk of stress urinary incontinence in previously irradiated patients [3,10].

The Optilume^®^ drug-coated balloon (DCB; Urotronic, Plymouth, MN, USA) was developed as a dual-action device combining mechanical dilation with localized delivery of paclitaxel, a microtubule-stabilizing agent that inhibits fibroblast proliferation and scar tissue formation [11,12]. Originally validated for anterior urethral strictures in the ROBUST trial program, Optilume received FDA clearance for recurrent bulbar strictures ≤3 cm in 2021, with five-year data demonstrating 71.7% freedom from reintervention [12,13]. Its application to posterior urethral stenoses, including BNS and VUAS, represents an off-label but increasingly explored frontier. This review synthesizes the current evidence on Optilume DCB for BNS and VUAS, compares outcomes between these entities, examines the influence of radiation therapy, and identifies future research priorities.

A structured literature search was conducted in PubMed/MEDLINE, Embase, and Scopus covering January 2020 to October 2025, using combinations of the terms “Optilume”, “drug-coated balloon”, “paclitaxel”, “bladder neck contracture”, “bladder neck stenosis”, “vesicourethral anastomotic stenosis”, “vesicourethral anastomotic stricture”, and “posterior urethral stricture”. Full-text English-language studies and major congress abstracts reporting at least five adult patients with BNS or VUAS treated with the Optilume DCB were considered; narrative reviews, non-human studies, and reports limited to anterior urethral strictures were excluded unless used to contextualize mechanism or foundational evidence.

## 2. Pathophysiological and Histological Distinctions

### 2.1. BNS Versus VUAS Versus Anterior Urethral Strictures

Anterior urethral strictures are characterized by spongiofibrosis-progressive fibrotic replacement of the corpus spongiosum that narrows the urethral lumen [8]. The histological hallmark is dense collagen deposition within an intact urethral wall with preserved surrounding tissue architecture. Common etiologies include trauma, instrumentation, infection, and lichen sclerosus [8].

BNS and VUAS fundamentally differ from anterior strictures in several respects. First, they occur at a surgically altered site lacking normal urethral wall architecture [3,4]. In BNS, fibrosis develops at the bladder neck following transurethral resection or ablation, where the smooth muscle has been disrupted and replaced by scar tissue [5,6]. Predisposing factors include extensive bladder neck resection, subclinical infection, and aggressive fulguration [5]. In VUAS, the stenosis forms at the surgical anastomosis between the bladder neck and membranous urethra after prostatectomy, where tissue ischemia from disrupted blood supply is a primary pathogenic driver [3,7]. The surrounding tissue in VUAS lacks the spongy vascular support of the corpus spongiosum, resulting in a relatively avascular fibrotic ring [3,7].

These differences have direct clinical implications. The avascular nature of VUAS may explain the consistently lower treatment success rates compared with BNS, as poor tissue perfusion limits both healing and drug penetration [7,9]. Ostad et al. reported freedom from secondary intervention of 87.5% for BNS versus only 40% for VUAS (*p* = 0.11), attributing this difference to ischemia at the anastomotic site [7]. Similarly, Jelisejevas et al. found comparable recurrence rates of approximately 34% for both membranous strictures and VUAS in their 53-patient series [14].

### 2.2. The Role of Radiation Therapy

**Figure 1:**
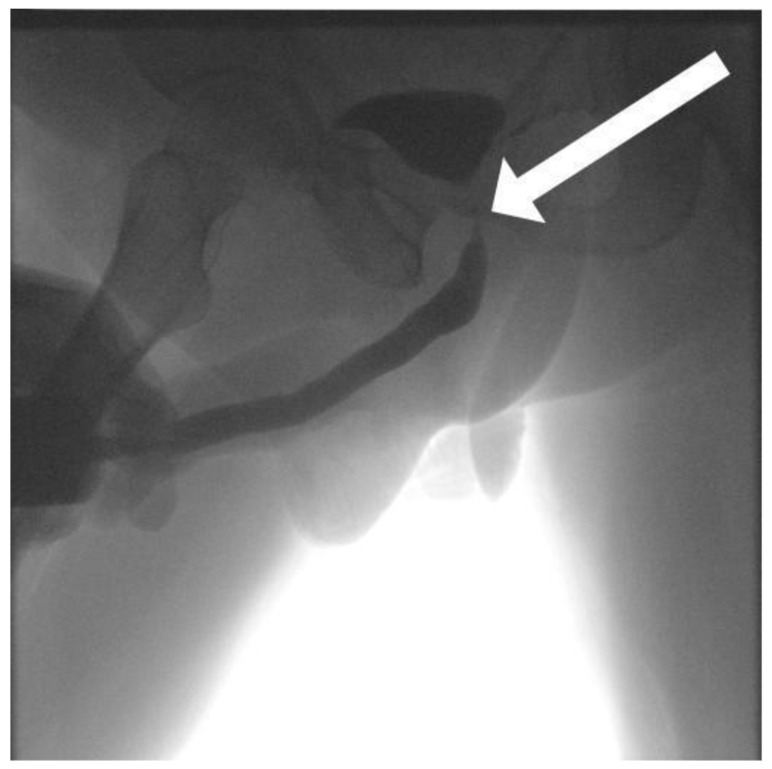
Cystourethrogram. The arrow indicates vesicourethral anastomotic stenosis following radiation therapy for prostate cancer.

**Figure 2:**
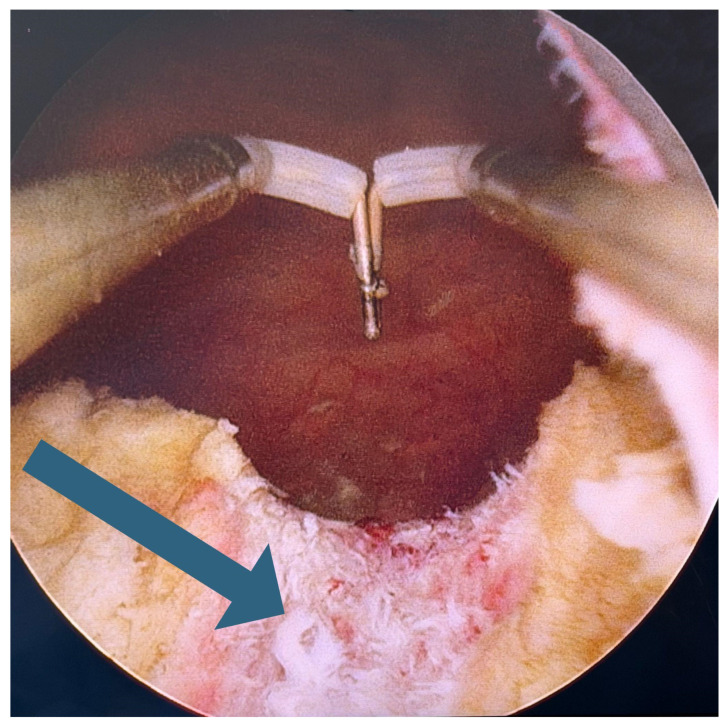
Urethroscopy. The arrow indicates ischemic urethral mucosa following radiation therapy for prostate cancer.

Radiation therapy introduces a distinct layer of pathophysiology to posterior urethral stenosis. Ionizing radiation causes direct DNA damage, endothelial injury, and progressive obliterative endarteritis, leading to tissue hypoxia, fibroblast proliferation, atrophy, and contraction [3,10]. These changes can be observed in cystourethrography (Figure 1) and urethroscopy (Figure 2). The incidence of radiation-induced posterior urethral stenosis (RIPUS) varies by modality: 2% following external beam radiotherapy (EBRT) alone, 4% after brachytherapy, and 11% with combined treatments, with cumulative rates at 10 years reaching 9.6%, 12%, and 19%, respectively [3,10].

Radiation-damaged tissue presents unique therapeutic challenges. Endoscopic treatment of RIPUS carries a 49% recurrence rate in brachytherapy patients at median 16-month follow-up [10]. Even urethroplasty, the gold standard for non-irradiated strictures with 80–95% success rates, achieves only 70–90% patency in irradiated patients, with 14.3% requiring repeat intervention and 19% developing post-operative stress urinary incontinence [3,10]. Dokter et al. found that 11 of 12 patients requiring reintervention after DCB had a history of pelvic radiation (*p* = 0.04), underscoring radiation as a significant negative predictor of DCB success [15]. Jelisejevas et al. confirmed prior pelvic irradiation as a statistically significant predictor of treatment failure (*p* = 0.047) [14].

Paradoxically, the anti-proliferative mechanism of paclitaxel may be particularly well-suited to radiation-induced fibrosis, as the drug directly targets the fibroblast proliferation pathway that drives post-radiation stenosis [10,11]. Ceballos et al. reported 81.1% freedom from repeat intervention in 37 patients with RIPUS treated with Optilume DCB, suggesting meaningful efficacy even in this challenging population [10]. Ostad et al. similarly observed 77.8% success in irradiated patients [7], and Hertz et al. reported 100% success in four patients with post-ablative therapy strictures at 6–14 months [16].

### 2.3. Procedural Considerations and Target Lesion Localization

**Figure 3:**
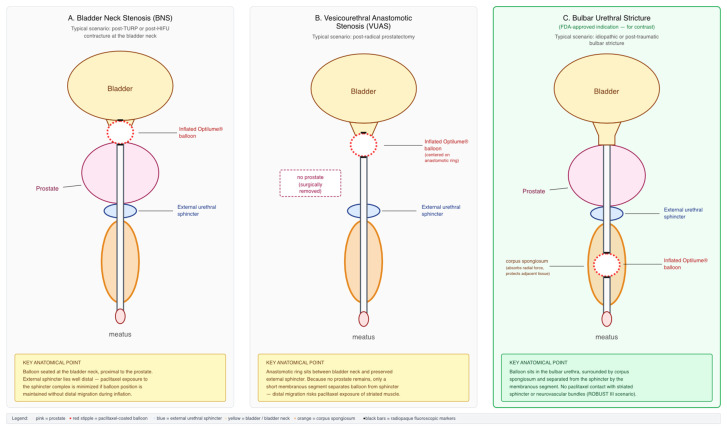
Schematic sagittal illustration of Optilume^®^ drug-coated balloon positioning for three posterior urethral targets: (**A**) bladder neck stenosis, with the balloon straddling the bladder neck and proximal prostatic urethra; (**B**) vesicourethral anastomotic stenosis after radical prostatectomy, with the balloon centered on the anastomotic line proximal to the preserved external urethral sphincter; and (**C**) membranous/bulbomembranous stricture, with the balloon positioned within the membranous urethra and taking care to avoid direct dilation of the sphincteric complex. Diagram created by the authors; not to scale.

Optilume DCB treatment for BNS and VUAS is performed under general or spinal anesthesia in the dorsal lithotomy position. After cystourethroscopy to confirm the length, location, and caliber of the stenosis, a hydrophilic guidewire is advanced across the lesion into the bladder under direct vision and fluoroscopic guidance. The 30 mm × 30 F paclitaxel-coated balloon catheter is then tracked over the wire until the radio-opaque balloon markers straddle the stenotic segment. The balloon is inflated to the manufacturer-recommended pressure for at least five minutes, during which mechanical dilation and elution of paclitaxel into the submucosal tissue occur concomitantly. Careful positioning is essential for bladder neck and vesicourethral anastomotic lesions because the external urethral sphincter lies immediately distal to the target in both conditions; excessive distal balloon migration can expose the sphincter mechanism to radial dilation force and, more importantly, to paclitaxel with unknown long-term effects on striated musculature and neural structures. Figure 3 schematically illustrates recommended catheter positioning for BNS, VUAS, and (for contrast) membranous/bulbomembranous strictures. After deflation, the balloon is withdrawn and a 16 F silicone Foley catheter is typically left in place for 48–72 h; this duration is adopted from the ROBUST protocols and has been used with minor institutional variation in most published BNS/VUAS series.

### 2.4. Device Differentiation: Optilume^®^ for BPH Versus Urethral Stenosis

Two Optilume^®^ products sharing the same paclitaxel coating but with distinct mechanical platforms are currently marketed. The Optilume BPH Catheter System is a two-catheter sequential device for prostatic urethral lobe separation. A pre-dilation balloon is first inflated to approximately 3 atm for 1 min to create a controlled anterior commissurotomy at the 12 o’clock position; it is then exchanged over the wire for a separate drug-coated balloon (diameter up to approximately 90 Fr; lengths 30, 35, 40, or 45 mm, chosen by transrectal ultrasound-measured prostatic urethral length), which is inflated at rated burst pressure for 5–10 min to deliver paclitaxel along the fresh incision edges.

The Optilume^®^ Urethral Drug-Coated Balloon is a single-balloon, over-the-wire, dual-lumen catheter with a tapered atraumatic tip, paclitaxel coating at 3.5 µg/mm^2^, and 18 Fr–30 Fr cystoscope compatibility. It is available in diameters of 18–36 Fr and lengths of 3, 4, and 5 cm; the 30 mm × 30 Fr configuration is most commonly reported in the posterior urethral literature. Pre-dilation with an uncoated balloon or cold-knife/Collings urethrotomy is performed in the same setting before DCB inflation. The device is used off-label for BNS and VUAS in all studies reviewed here, having received FDA approval only for recurrent anterior urethral strictures ≤3 cm.

## 3. Evidence for Optilume DCB in BNS and VUAS

### 3.1. Foundational Evidence from Anterior Strictures

The ROBUST trial program established the efficacy of Optilume DCB for anterior urethral strictures. ROBUST I demonstrated 71.7% freedom from reintervention at five years, with sustained Qmax improvement from 5.0 to 19.9 mL/s and IPSS reduction from 25.2 to 7.2 [12,13]. The pivotal ROBUST III randomized controlled trial (*n* = 127) showed 77.8% freedom from repeat intervention at two years in the DCB arm versus 23.6% in the control arm (*p* < 0.001) [17]. A systematic review and meta-analysis by Estaphanous et al. encompassing 457 patients reported a pooled recurrence-free rate of approximately 81%, with mean IPSS reduction of 13 points and Qmax improvement of 10.1 mL/s [11]. These results formed the rationale for exploring DCB in posterior stenoses.

### 3.2. BNS-Specific Outcomes

Several studies have specifically reported outcomes for BNS following Optilume DCB. Tosev et al. treated 16 patients with BNS/VUAS (50% post-TURP, 43.8% post-radical prostatectomy) and reported 100% freedom from repeat intervention and anatomic recurrence at one year, with IPSS improving from 28.1 to 14.6 (*p* < 0.001, Cohen’s d = 1.06) [9]. Sitharthan et al. described the first reported use of Optilume for refractory post-TURP BNS, achieving catheter-free status with minimal LUTS at six-month follow-up after four prior failed endoscopic treatments [5]. Ostad et al. reported 87.5% freedom from secondary intervention for BNS in their 13-patient series [7]. Cretì et al. included two bladder neck stenosis patients among 35 treated, observing 91.4% overall success at six months with Qmax improvement from 10.2 to 21.6 mL/s (*p* < 0.001) [18].

### 3.3. VUAS-Specific Outcomes

VUAS outcomes with Optilume DCB have been more heterogeneous. Ostad et al. reported only 40% freedom from secondary intervention for VUAS compared with 87.5% for BNS, though this did not reach statistical significance (*p* = 0.11) [7]. By contrast, Jelisejevas et al. found 66.6% of 18 VUAS patients free from recurrence at median 13.3 months [14]. Pepe et al. treated seven patients with vesicourethral stenosis following radical prostatectomy as part of a 20-patient series, reporting 100% freedom from reintervention at median 18 months with IPSS improving from 27 to 10 and Qmax from 5 to 10 mL/s at 12 months [19].

### 3.4. Comparative and Large Cohort Data

The first comparative study of Optilume DCB versus standard endoscopic treatment for posterior urethral stenosis was reported by Berg et al. in a retrospective cohort of 141 patients (65 DCB, 76 standard treatment; 82% VUAS, 18% BNS) [20]. Recurrence-free survival was significantly improved in the DCB group (*p* = 0.013), with multivariate analysis demonstrating a 60% reduced risk of recurrence (HR 0.40, 95% CI 0.19–0.87, *p* = 0.021). Functional outcomes included Qmax improvement from 9 to 22 mL/s (*p* = 0.001) and PVR reduction from 55 to 0 mL (*p* < 0.001) [20]. Notably, stenosis localization (BNS versus VUAS) was not a significant predictor of recurrence-free survival in this series [20].

Patel et al. reported the largest real-world cohort to date from the TURNS database, including 59 patients with posterior urethral stenoses (44 BNS/VUAS, 11 membranous, 5 prostatic) [21]. One-year functional recurrence-free survival was 75.8% and anatomical recurrence-free survival was 59.4% for posterior stenoses, compared with 78.4% and 66.4% for anterior strictures, respectively [21]. Dokter et al. analyzed 53 men with posterior urethral strictures and bladder neck contractures, reporting 77.4% freedom from surgical reintervention at median 15.9 months, with pelvic radiation significantly predicting failure (*p* = 0.04) [15]. 

The results of notable studies are summed up in Table 1.

## 4. Discussion

### 4.1. Mechanism of Action and Rationale for Posterior Stenoses

Paclitaxel, a microtubule-stabilizing agent originally repurposed from vascular drug-coated balloons, inhibits smooth muscle cell migration and fibroblast proliferation—the principal cellular drivers of stricture recurrence [11,12]. The Optilume device delivers paclitaxel directly to the dilated stricture site, where the drug coating adheres to tissue as visible flakes, maximizing local concentration while minimizing systemic exposure [12,22]. This dual-action mechanism (mechanical dilation plus anti-fibrotic drug delivery) is theoretically well-suited to BNS and VUAS, where the recurrence is fundamentally driven by fibrotic contracture rather than spongiofibrosis [3,4,9].

However, the avascular fibrotic environment of VUAS may limit paclitaxel penetration and retention, potentially explaining the lower success rates compared with BNS, where blood supply, although compromised, is generally better preserved [3,7]. Alhamdani et al. demonstrated that meticulous technique—including pre-treatment incision, minimal irrigation, and omitting routine post-operative catheterization to maximize drug contact time—may optimize outcomes, achieving 76% re-treatment-free survival at 30 months in a cohort with a mean of 7.7 prior procedures [22].

### 4.2. BNS Versus VUAS: A Comparative Perspective

The emerging literature consistently suggests that BNS responds more favorably to DCB treatment than VUAS. Ostad et al. observed freedom from secondary intervention rates of 87.5% versus 40% for BNS and VUAS, respectively [7]. This differential likely reflects the distinct pathophysiological milieu: BNS involves fibrotic contracture of a surgically disrupted but still relatively vascularized bladder neck, whereas VUAS represents scarring at an inherently ischemic anastomotic site [3,7]. However, Berg et al. did not find stenosis localization to be a significant predictor of recurrence-free survival in their comparative study, suggesting that with adequate sample sizes and refined technique, outcomes may converge [20]. Tosev et al. further reported 100% success in a mixed BNS/VUAS cohort at 12 months, though their sample included 50% post-TURP patients who may represent a more favorable phenotype [9].

### 4.3. Radiation as a Modifier of Treatment Outcomes

Radiation exposure consistently emerges as the most significant negative prognostic factor for DCB treatment of posterior stenoses. Dokter et al. found that 11 of 12 reintervention patients had prior pelvic radiation (*p* = 0.04) [15], while Jelisejevas et al. identified radiation as a significant predictor of failure (*p* = 0.047) [14]. Yet the story is nuanced: Ceballos et al.’s multi-institutional series of 37 patients with exclusively radiation-induced posterior urethral stenosis achieved 81.1% freedom from repeat intervention [10], and Ostad et al. observed 77.8% success specifically in irradiated patients [7]. These findings suggest that while radiation confers additional risk, DCB may still offer meaningful benefit over conventional endoscopic management in irradiated patients, for whom traditional approaches carry recurrence rates exceeding 49% [3,10].

### 4.4. Safety Profile and Continence Considerations

Across all reviewed studies, the safety profile of Optilume DCB for posterior stenoses has been favorable. Complications have been predominantly Clavien-Dindo grade I, including transient hematuria (5–15%), acute urinary retention (3–5%), and urinary tract infection (3–6%) [7,9,10,14,15,20]. No studies reported de novo incontinence attributable to the device [9,14,20]. Jelisejevas et al. specifically noted that two patients with pre-existing post-prostatectomy incontinence experienced symptom recurrence after successful dilation, representing unmasking of pre-existing incontinence rather than a device-related complication [14]. Erectile function has been preserved or improved across studies reporting sexual outcomes, with Cretì et al. demonstrating statistically significant IIEF-5 improvement (*p* = 0.012) [18] and Pepe et al. reporting IIEF-5 improvement from 7 to 16 at 12 months [19].

### 4.5. Pre-Treatment Imaging Assessment

A critical gap in the current management of BNS and VUAS is the lack of standardized pre-treatment imaging to characterize fibrosis severity, tissue vascularity, and stricture morphology before qualifying patients for DCB treatment. In anterior urethral stricture disease, Frankiewicz et al. systematically reviewed the role of magnetic resonance imaging (MRI) and demonstrated that MRI can reliably visualize periurethral spongiofibrosis on T2-weighted sequences, where fibrotic tissue appears as low signal intensity replacing the normal high-signal corpus spongiosum [23]. MRI-assessed spongiofibrosis length (SFL) has been shown to exceed stricture length measured by retrograde urethrography (14.9 vs. 7.9 mm, *p* < 0.0001), and SFL independently predicts surgical complexity, including operation time (*p* = 0.02) and blood loss (*p* = 0.04) [23,24]. Importantly, MRI overcomes the principal limitation of retrograde urethrography—its inability to assess periurethral tissue quality—and offers a more objective alternative to sonourethrography (SUG), which remains highly operator-dependent [23].

Despite these advantages for anterior strictures, no published studies have applied MRI-based fibrosis assessment to BNS or VUAS. Our PubMed search yielded zero results for MRI fibrosis grading in the context of vesicourethral anastomotic stenosis or bladder neck contracture. Mac Curtain et al. noted that MRI may be useful in evaluating complex or previously irradiated BNS/VUAS patients, but no formal grading system has been validated for these indications [3]. This represents a significant research opportunity, as the degree and extent of periurethral fibrosis could theoretically predict DCB treatment response and guide patient selection—distinguishing patients with limited, potentially DCB-responsive fibrosis from those with extensive obliterative scarring more appropriate for reconstructive surgery.

An alternative imaging approach for posterior stenoses is transrectal ultrasonography (TRUS). While sonourethrography has been established as a tool for assessing spongiofibrosis in anterior urethral strictures by evaluating the echogenicity and thickness of periurethral tissue, its application is anatomically limited to the anterior urethra where a penile or perineal probe can be applied [23]. For posterior stenoses at the bladder neck or vesicourethral anastomosis, TRUS offers an analogous acoustic window. Pepe et al. demonstrated the feasibility of ultrasound-guided DCB treatment, using a transrectal biplanar probe to evaluate vesicourethral anastomotic stenosis and a linear probe for anterior strictures, allowing real-time assessment of stenosis length, corpus spongiosum echopattern, and dilation adequacy [19]. Their technique—the first reported ultrasound-guided Optilume procedure—eliminated fluoroscopic radiation exposure while providing direct tissue characterization [19]. Extending this approach, TRUS-based fibrosis grading of the periurethral tissue at the bladder neck and anastomotic site could serve as a non-invasive tool for predicting DCB outcomes in BNS and VUAS, analogous to how SUG-based spongiofibrosis assessment informs treatment planning in anterior strictures.

### 4.6. Limitations of the Current Evidence

Several important limitations must be acknowledged. Firstly, the vast majority of studies are retrospective and single-center with small sample sizes and short follow-up (median 5.7–22.9 months) [7,9,10,11,14,15,19,20,21]. Only the ROBUST III trial represents level I evidence, and it excluded posterior stenoses [17]. Secondly, heterogeneity in inclusion criteria, variable follow-up windows (typically 3–24 months), surgical technique, balloon sizing, and non-standardized success definitions that mix freedom from reintervention, objective patency (≥17 F passage), and symptom-based endpoints (Qmax, IPSS) limits cross-study comparison [8,21]. Comparator arms are usually absent or historical. Publication bias toward positive single-center experiences is likely, and reported effect sizes should therefore be treated as provisional upper-bound estimates rather than definitive efficacy measures. Thirdly, there is no head-to-head comparison with urethroplasty, which remains the gold standard with 80–95% success for non-irradiated strictures [3,8]. Patel et al.’s real-world TURNS data demonstrated lower success rates than the controlled ROBUST III trial (66.4% vs. 74.6% anatomical recurrence-free survival at one year), highlighting the gap between trial and practice settings [21]. Finally, the use of Optilume for BNS and VUAS remains off-label, and cost-effectiveness data specific to posterior stenoses are lacking [4,8].

### 4.7. A Proposed Patient Selection Framework

**Figure 4:**
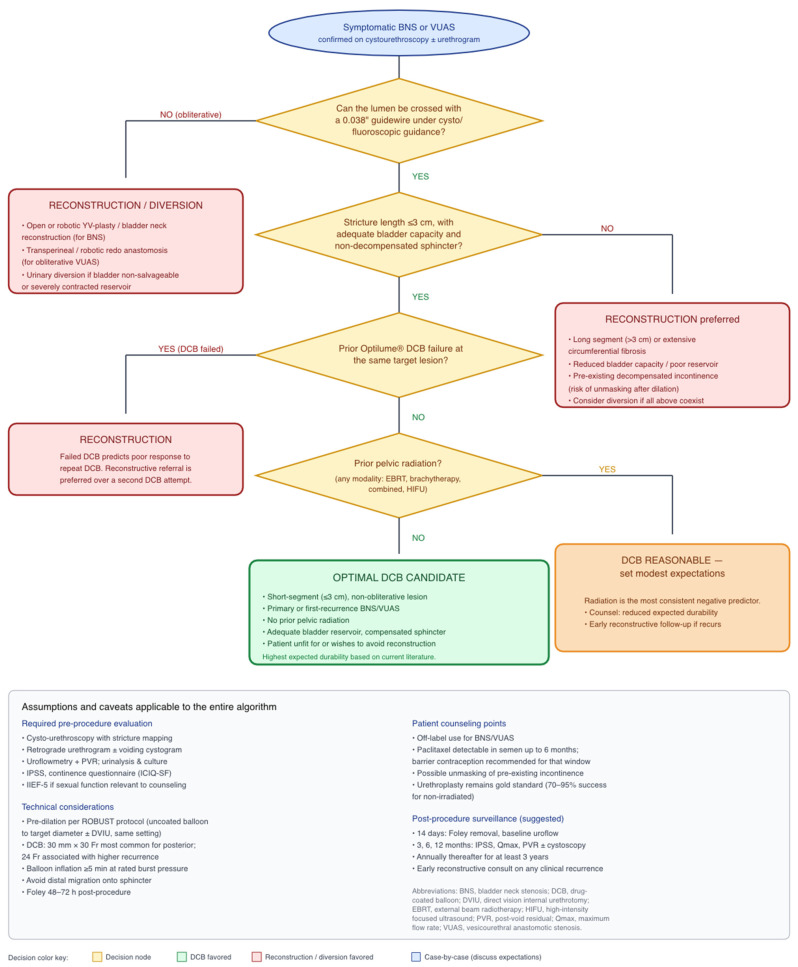
Proposed decision algorithm for Optilume^®^ DCB versus standard endoscopic management versus urethroplasty in BNS and VUAS. Key decision nodes are stricture length, prior pelvic radiation, number of prior endoscopic treatments, and fitness for reconstructive surgery. Figure developed by the authors from synthesized evidence; prospective validation is required.

Drawing the available evidence together, a practical selection framework can be offered while acknowledging that every component requires prospective validation. In broad terms, Optilume DCB appears most attractive for (i) primary or first-recurrence BNS or VUAS, (ii) lesions ≤2 cm in length, (iii) patients without prior pelvic radiation or with well-compensated post-radiation tissue, and (iv) patients who are unfit for or actively wish to avoid open/robotic reconstruction. Conversely, patients with long-segment (>3 cm) or obliterative lesions, densely irradiated tissue with poor bladder capacity or reservoir function, or documented failure of two or more prior endoscopic treatments including a previous DCB are probably better served by early reconstructive referral. The algorithm summarized in Figure 4 stratifies candidates by these variables and is intended as a clinical starting point, not a guideline.

### 4.8. Device Availability, Training, and Economic Context

Two considerations that materially affect real-world adoption warrant brief mention. First, geographic availability of the Optilume urethral DCB remains uneven: the device is FDA-cleared and reimbursed in the United States, has CE mark but variable national coverage across Europe, and is not yet routinely available in large parts of Asia, Africa, and Latin America. Second, while the procedure itself is technically straightforward for a practicing endourologist, correct identification of the target lesion—particularly distinguishing true VUAS from bladder-neck involvement or from bulbomembranous recurrence—benefits from combined cysto-urethroscopy and fluoroscopy, and institutions new to the device should plan a short proctored learning period. From an economic standpoint, the acquisition cost of the DCB (approximately 1.5–3.5 times that of a standard balloon dilator, depending on market) is non-trivial; its cost-effectiveness will ultimately depend on durability beyond the first year and on reduced downstream reconstruction rates, neither of which is yet definitively established for the posterior urethra. Formal cost-utility analyses, ideally from national health-system datasets, are a priority.

## 5. Future Research Directions

Several priorities emerge for advancing the evidence base. Prospective randomized trials comparing Optilume DCB with standard endoscopic management and urethroplasty specifically for BNS and VUAS are critically needed [4,8,20]. Stratified analyses by radiation status, stricture etiology (post-TURP vs. post-prostatectomy vs. post-radiation), and number of prior treatments would inform patient selection [8,14,15]. The optimal technical protocol—including balloon sizing, inflation duration, pre-dilation strategy, and post-procedure catheterization—remains undefined for posterior stenoses and warrants standardization [8,22]. Biomarker studies examining tissue paclitaxel levels and histological response in posterior urethral tissue could elucidate why VUAS responds less favorably than BNS [7]. Finally, long-term follow-up beyond five years and cost-effectiveness analyses are essential to justify broader adoption [8,12,13].

## 6. Conclusions

Optilume DCB represents a promising minimally invasive treatment for BNS and VUAS, offering a mechanistically rational approach that combines mechanical dilation with targeted anti-fibrotic drug delivery. The current evidence, though predominantly retrospective and of moderate quality, suggests freedom from reintervention rates of 65–100% for BNS and 40–81% for VUAS at short-to-intermediate follow-up. BNS appears to respond more favorably than VUAS, supposedly reflecting differences in tissue vascularity and fibrotic biology. Prior radiation therapy seems to be the most consistent negative prognostic factor but does not preclude meaningful benefit. The safety profile is favorable, with no de novo incontinence and preserved sexual function. These conclusions should be interpreted cautiously: comparisons against standard endoscopic management or urethroplasty are almost entirely indirect, success definitions vary across studies, and long-term (≥5-year) data for the posterior urethra are not yet available. Until adequately powered, prospective, ideally randomized comparative trials with standardized outcome definitions, stratification by radiation status and prior treatment history, and dedicated cost-effectiveness analyses are reported, the Optilume DCB should be regarded as a reasonable off-label option in selected patients with short-segment, non-obliterative BNS or VUAS who are unfit for or wish to avoid reconstruction, rather than as an established first-line therapy.

## Figures and Tables

**Table 1:** Summary of studies reporting Optilume^®^ drug-coated balloon outcomes for bladder neck stenosis (BNS) and vesicourethral anastomotic stenosis (VUAS).

Study *	Number of Patients (BNS/VUAS)	Study Design (LoE)	Stenosis Etiology	Prior Treatments	Follow-Up	FFRI (%) at Follow-Up	IPSS (Pre → Post) [9]	Qmax (Pre → Post)	Complications	De Novo UI
Tosev et al. 2025 [9]	16 (8 BNS, 7 VUAS, 1 mixed)	Retrospective single-center	100% iatrogenic etiology	50% post-TURP, 43.8% post-RP, 6.2% GreenLight; 43.8% prior pelvic RT; 56.2% had 1; range 1–15	12 mo	100% (combined BNS/VUAS)	28.1 → 14.6 (*p* < 0.001)	NR	NR	0%
Ostad et al. 2025 [7]	13 (8 BNS, 5 VUAS)	Retrospective single-center	NR	92.3% prostate cancer; 69.2% prior RT; Median 1 (IQR 1–4)	Median 464 d	69.2% (n = 9) for the entire cohort and 77.8% (n = 7) for radiated patients	NR	NR	No major complications	NR
Berg et al. 2025 [20]	141 (65 DCB (53 VUAS 12 BNS), 76 ST)	Retrospective cohort comparative single-center	NR	DCB: 82% post-RP/cystectomy, 18% TURP/enucleation; 15% RT. ST: 67% post-RP/cystectomy, 32% TURP; 28% RT; No of prior treatments: Median 1 (IQR 0–3) DCB	Median 13 mo	DCB vs. ST: HR 0.40 (95% CI 0.19–0.87, *p* = 0.021)	NR	9 → 22 mL/s (*p* = 0.001)	DCB:4.6% (2 UTI, 1 AUR); ST: none	0%
Ceballos et al. 2025 [10]	56 (bulbomembranous)	Retrospective multi-center	100% prior pelvic RT	55.4% had prior intervention; mean 1.25 dilations (SD2.57)	Mean 179.4 d (SD 134.9)	81.1% (30/37)	NR	NR	10.7% (6/56): AUR 3 (5.4%), clot retention 2 (3.3%), UI 1 (1.7%); none > CD IIIb	NR
Jelisejevas et al. 2025 [14]	53 (35 membranous, 18 VUAS)	Retrospective single-center	69.8% Iatrogenic 11.3% Congenital 9.4%, Idiopathic 7.6%, Traumatic, 1.9% Lichen sclerosus; 32% prior RT	Median 2.5 (IQR 1–7.25)	Median 13.3 mo	Membranous 65.6%; VUAS 66.6%	NR	NR	CD I 3.8% (2/53): 1 urethral pain 1.9%, 1 urethral bleeding 1.9%	0%
Dokter et al. 2025 [15]	53 (15 BNS, 38 membranous or prostatic US)	Retrospective single-center	NR	Mean number of treatments 2,4; 37 (70%): 6 (30%) prostatectomy, 35 (66%) pelvic RT, 10 (19%) both prostatectomy and RT, 10 (19%) TURP	Median 15.9 mo	77.4%	NR	NR	20,75% (11/53): gross hematuria (6/11), urinary retention (4/11), UTI (3/11)	NR
Patel et al. 2026 [21]	319 (260 AUS, 59 PUS (43 BNS/VUAS, 11 membranous, 5 prostatic))	Retrospective multi-center	5.0% External trauma, 28.1% Idiopathic, 12.3% Internal trauma, 25.6% Recurrent stricture, 22.4% Radiation, 0.9% Infectious/Inflammatory, 2.5% Hypospadias repair, 3.2% Lichen sclerosus	61.1% prior DVIU/dilation; 27.3% prior urethroplasty; 1.3% prior bladder neck repairs	Median 5.7 mo (IQR 3–12)	70.5% (95% CI 55.4–89.8)	NR	NR	20/319 (6.3%): AUR 6, LUTS 4, UI 3, hematuria 3, UTI/sepsis 3	NR
Pepe et al. 2025 [19]	31 (11 bulbar US, 13 AUS, 7 VUAS)	Retrospective, single-center	35% post-RP	100% endoscopic (19 endoscopic urethrotomy, 1 urethroplasty for hyospadias); 15% salvage RT (3)	Median 18 mo	71.7%	27 → 10 (12 mo)	5→10 mL/s (12 mo)	NR	NR

Abbreviations: AUR, acute urinary retention; AUS, anterior urethral stenosis; BNS, bladder neck stenosis; CD, Clavien–Dindo; CI, confidence interval; DCB, drug-coated balloon; DVIU, direct vision internal urethrotomy; FFRI, freedom from reintervention; HR, hazard ratio; IQR, interquartile range; IPSS, International Prostate Symptom Score; LoE, level of evidence; LUTS, lower urinary tract symptoms; mo, months; NR, not reported; PUS, posterior urethral stenosis; Qmax, maximum urinary flow rate; RP, radical prostatectomy; RT, radiotherapy; SD, standard deviation; ST, standard treatment; TURP, transurethral resection of the prostate; UI, urinary incontinence; US, urethral stricture; UTI, urinary tract infection; VUAS, vesicourethral anastomotic stenosis. * The results of the studies were cited with the highest possible granulation. When the data for BNS/VUAS groups were not reported, the results for the entire examined cohorts were used.

## Data Availability

No new data were created or analyzed in this study.

## Abbreviations

The following abbreviations are used in this manuscript:

anat	anatomical
AUR	acute urinary retention
AUS	anterior urethral stenosis
BNS	bladder neck stenosis
BPH	benign prostatic hyperplasia
CD	Clavien-Dindo
CI	confidence interval
d	days
DCB	drug-coated balloon
DVIU	direct vision internal urethrotomy
EBRT	external beam radiotherapy
FDA	Food and Drug Administration
FFRI	freedom from reintervention
func	functional
HIFU	high-intensity focused ultrasound
HR	hazard ratio
IIEF-5	International Index of Erectile Function-5
IPSS	International Prostate Symptom Score
IQR	interquartile range
LoE	level of evidence
LUTS	lower urinary tract symptoms
mo	months
MRI	magnetic resonance imaging
NR	not reported
PUS	posterior urethral stenosis
PVR	post-void residual
Qmax	maximum urinary flow rate
RARP	robot-assisted radical prostatectomy
RIPUS	radiation-induced posterior urethral stenosis
RP	radical prostatectomy
RT	radiotherapy
SD	standard deviation
SFL	spongiofibrosis length
ST	standard treatment
SUG	sonourethrography
TRUS	transrectal ultrasonography
TURNS	Trauma and Urologic Reconstructive Network of Surgeons
TURP	transurethral resection of the prostate
UI	urinary incontinence
US	urethral stricture
UTI	urinary tract infection
VUAS	vesicourethral anastomotic stenosis
